# Supplementary material for: Cycles of protein condensation and discharge in nuclear organelles studied by fluorescence lifetime imaging
Source: Nat Commun. 2019 Jan 28;10:455. doi: 10.1038/s41467-019-08354-3 (PMC6349932; doi:10.1038/s41467-019-08354-3)
Supplement: Supplementary file 1 — Supplementary Information [file 41467_2019_8354_MOESM1_ESM.pdf]

Supplementary Figures

a

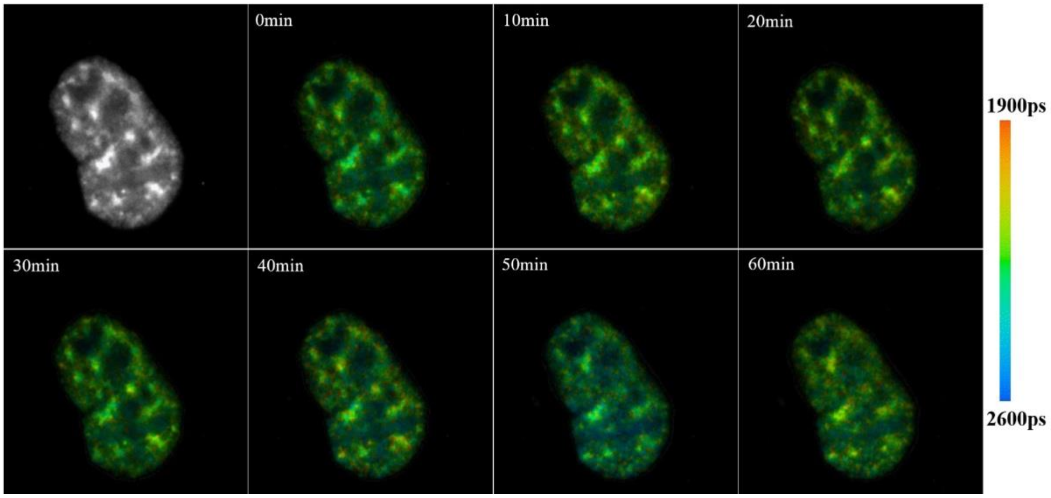

b

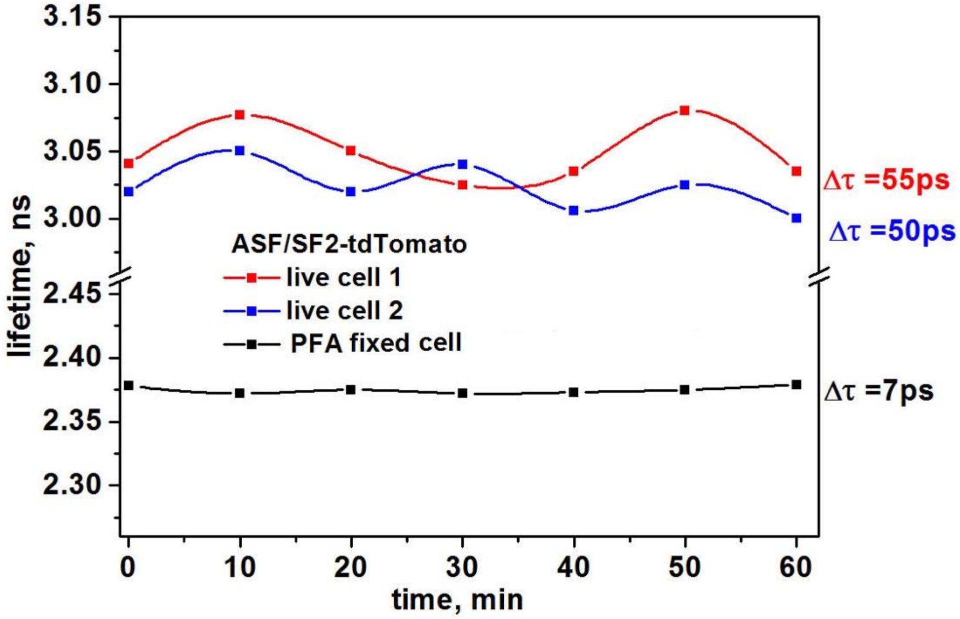

Supplementary Figure 1. Monitoring ASF/SF2-tdTomato Fluorescence Lifetimes in Live and Fixed Cells. *(**a**) FLIM images of ASF/SF2-tdTomato acquired in formaldehyde (PFA) fixed cells with 10 min intervals. Cells were kept at 37°C. (**b**) Charts with the fluorescence lifetime of ASF/SF2-2-tdTomato values measured in live and fixed cells, as indicated.*

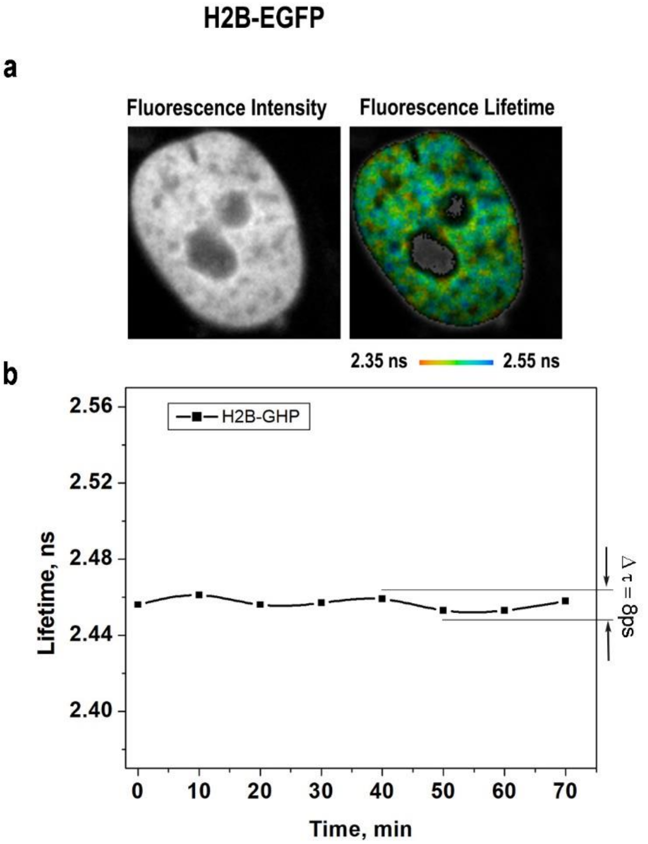

Supplementary Figure 2. Monitoring of Fluorescence Lifetime of histone H2B-EGFP.
*(**a**) The fluorescence intensity image and fluorescence lifetime images of histone H2B-EGFP in live cultured cell. (**b**) Averaged fluorescence lifetimes measured with 10 min intervals. The fluorescence lifetime variations were within 5-15 picoseconds.*

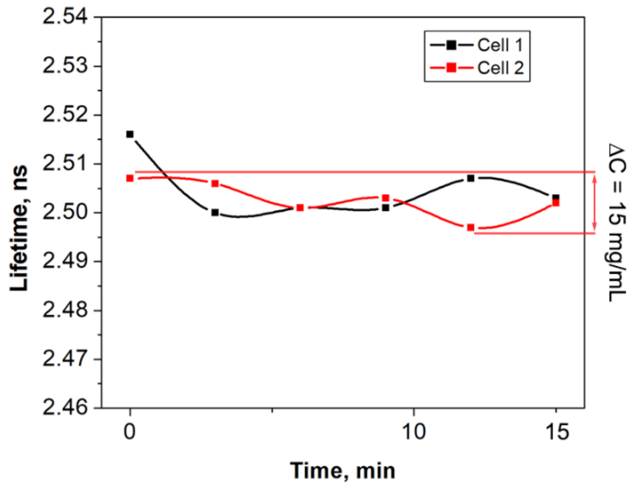

Supplementary Figure 3. Monitoring the ASF/SF2-EGFP fluorescence lifetime in the nuclear speckles of live cells at room temperature. *Plots show the fluorescence lifetime data and corresponding range of protein concentrations for two representative cells.*

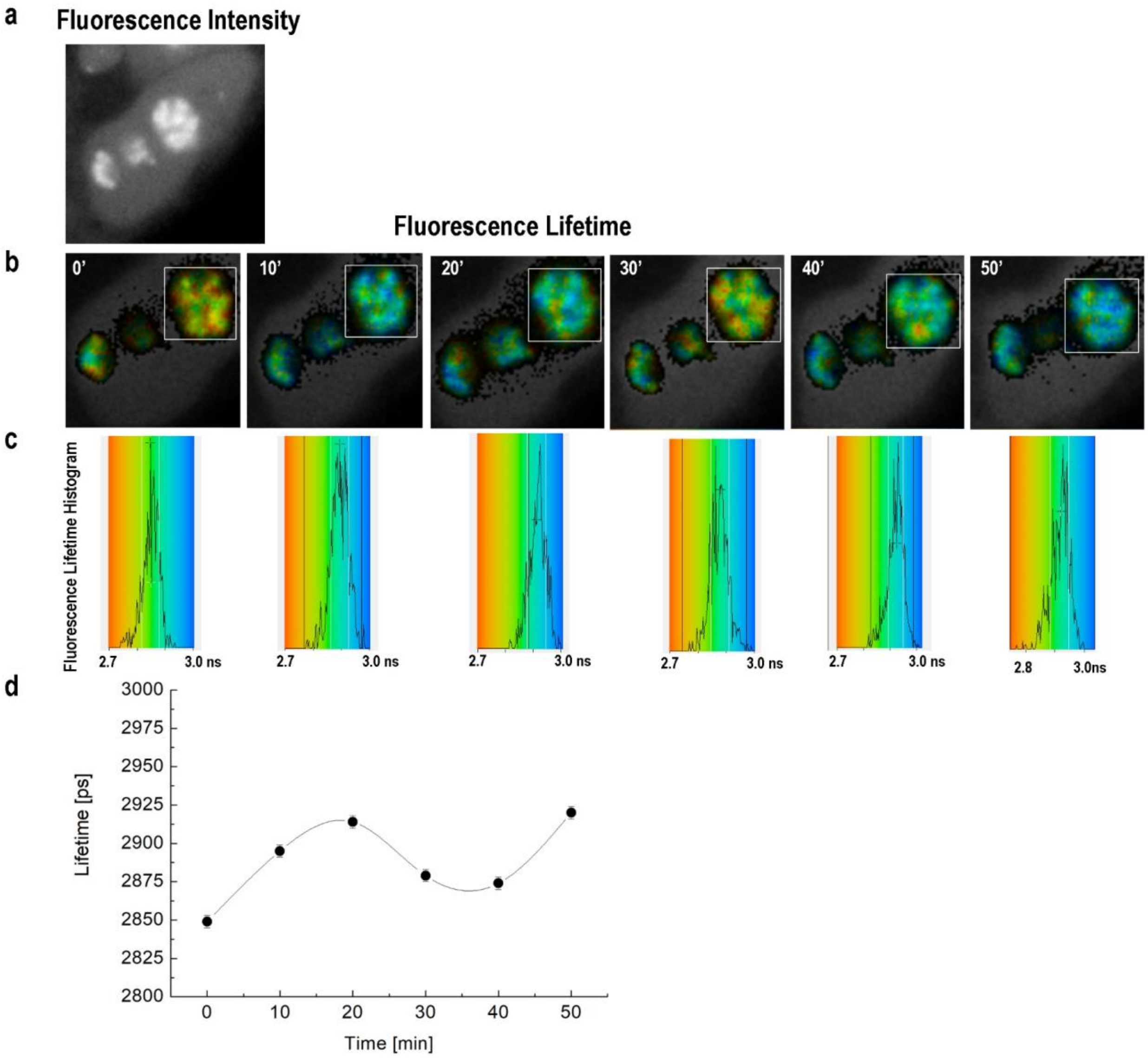

Supplementary Figure 4. Monitoring the tdTomato-Fibrillarin fluorescence lifetime in live cell nucleoli.

(a) *Fluorescence Image of tdTomato-Fibrillarin**.** **(b)** FLIM images acquired with 10 min intervals of the cell shown in (a); the largest nucleolus is highlighted by white boundaries. (**c**) Fluorescence lifetime histograms from the nucleolus selected in (b). (**d**) Fluctuations of the fluorescence lifetime in the selected nucleolus*

.

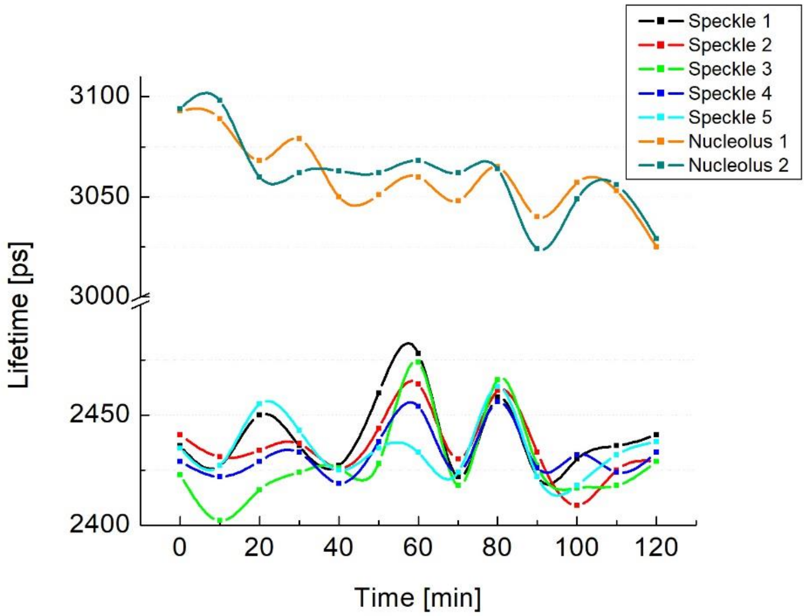

***Supplementary Figure 5*. Synchronous fluctuations of the fluorescence lifetimes in different nuclear organelles of the same cell**. Cells expressing eiser Fibrillarin-tdTomato or ASF/SF2-EGFP were monitored by FLIM. Plots show fluctuations of the fluorescence lifetimes in different nuclear speckles of the same cell, and in different nucleoli of the same cell, as indicated.

(a)

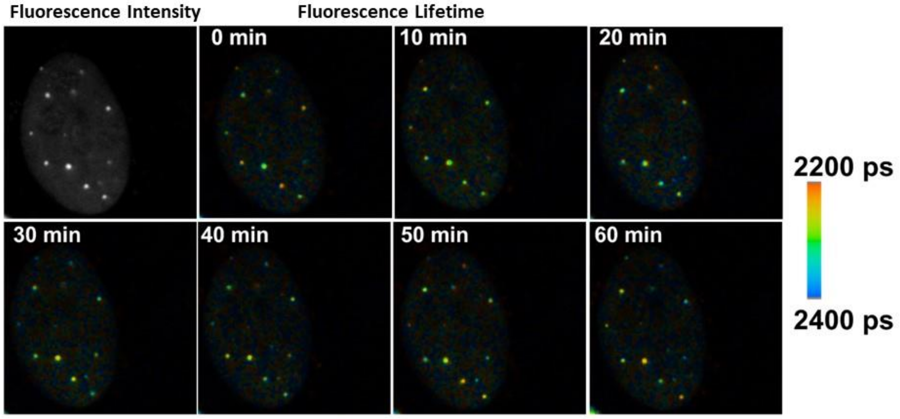

(b)

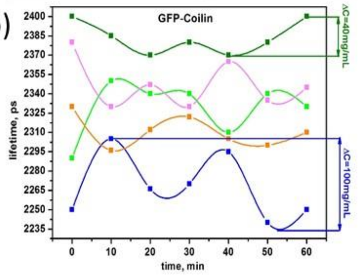

(c)

**Cell1**	ΔC = 70 mg/mL
**Cell2**	ΔC = 100 mg/mL
**Cell3**	ΔC = 40 mg/mL
**Cell4**	ΔC = 60 mg/mL
**Cell5**	ΔC = 35 mg/mL

Supplementary Figure 6. Cyclic Changes in Absolute Concentrations of Proteins in Cajal Bodies.
*(**a**) Upper left panel represents fluorescence intensity image of coilin-EGFP in live cultured cell, other panels demonstrate fluorescence lifetime data with 10 min intervals. (**b**) Fluorescence lifetimes were averaged for five cells and plotted over the time. (**c**) ΔC demonstrates amplitude in the protein concentrations changes per cell shown in charts on (b).*

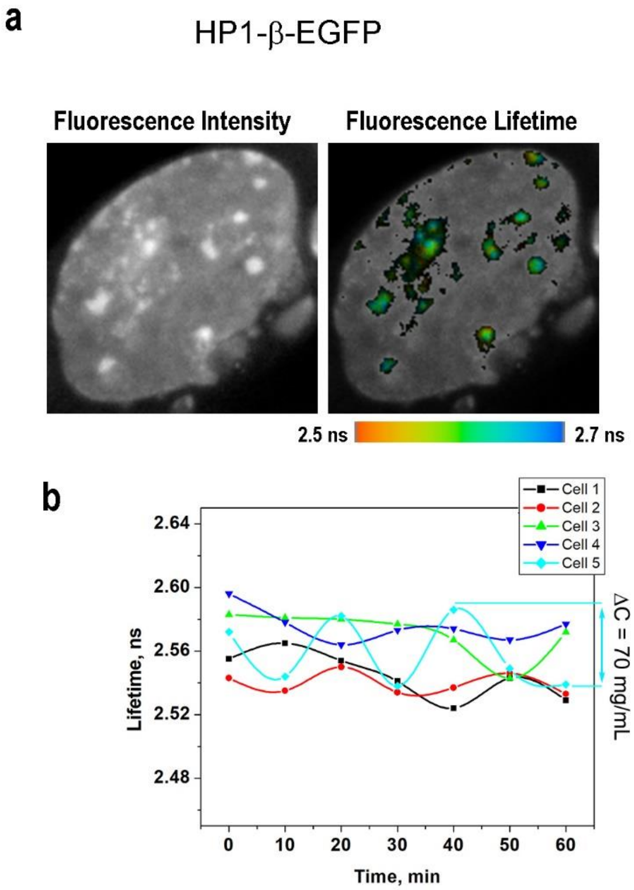

Supplementary Figure 7. Cyclic Changes of Proteins Absolute Concentrations in HP1-β **heterochromatin *domains***. *(**a**) Fluorescence intensity and fluorescence lifetime images of* HP1-β-EGFP *in live cultured cell. (**b**) Charts show the fluorescence lifetimes of* HP1-β-EGFP *acquired with 10 min intervals in single cells. For each time point, lifetime data were averaged in all* HP1-β domains in the cell. *ΔC demonstrates the amplitude in the protein concentrations changes per single cell.*

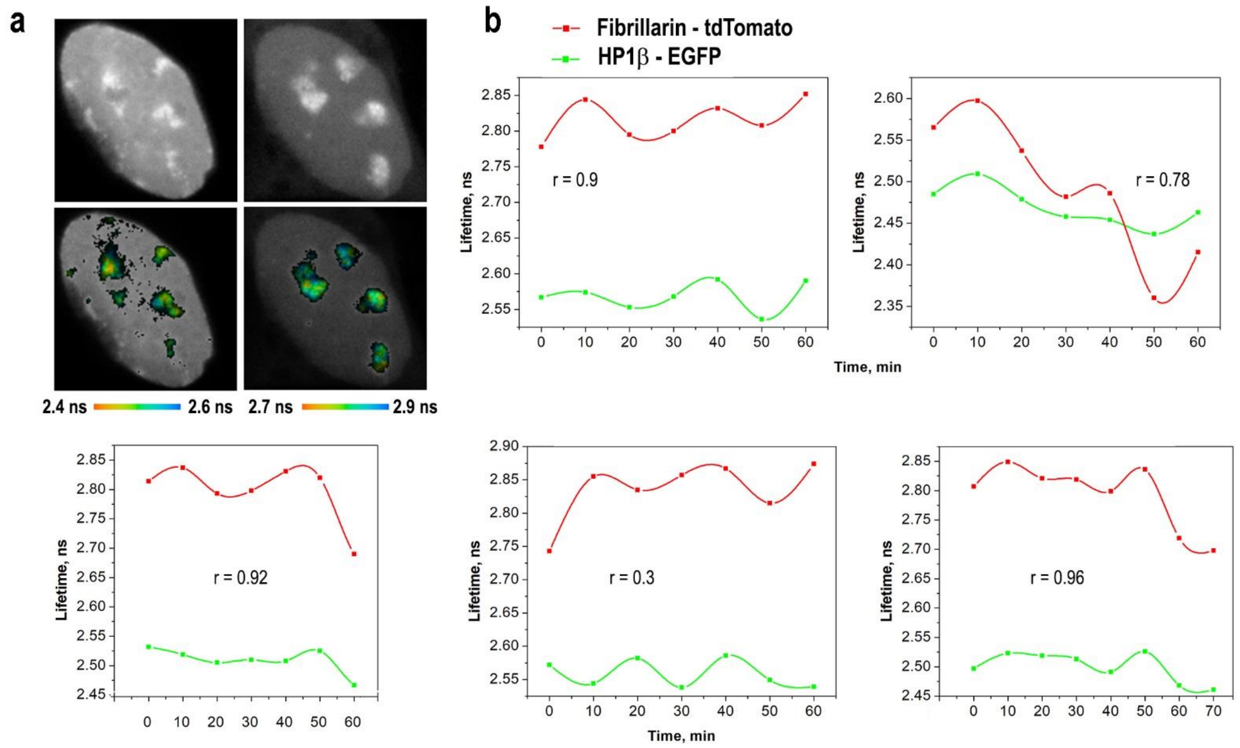

***Supplementary Figure* 8. *Correlation of fluorescence lifetimes in nucleoli and HP1-β heterochromatin domains*.** *(**a**) Simultaneous monitoring of fluorescence lifetimes of HP1-β - EGFP* and *Fibrillarin-tdTomato in heterochromatin domains and nucleoli of the same cells. (**b**) Fluctuations of fluorescence lifetimes of Fibrillarin-tdTomato and HP1-β - EGFP* in the same cells*. The Pearson correlation coefficients (r) on the charts indicate significant correlation between fluorescence lifetime changes for each cellular data set.*

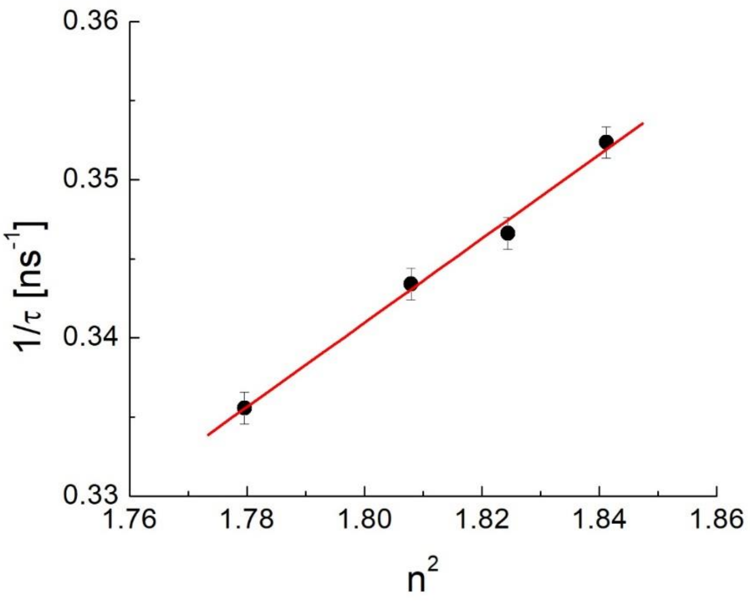

Supplementary Figure 9. Dependence of fluorescence lifetime of EGFP on the refractive index in BSA/PBS solutions.

GFP was dissolved in PBS, and in 100, 150 and 200 mg/ml solutions of BSA in PBS. Fluorescent lifetime decays for EGFP in these solutions were measured. Inverse fluorescence lifetime (1/τ) is plotted against the square of the refractive index (n). Error bars are the Std. dev. (n=15 for each calibration point).
